# High-temperature sintered 3D-printed alumina as mechanically robust supports for MOF catalysis

**DOI:** 10.1039/d5ma01028d

**Published:** 2025-10-10

**Authors:** Flora Schöfbeck, Tanja Eder, Wenyi Zeng, Dominik Brouczek, Martin Schwentenwein, Youven Benseghir, Michael R. Reithofer, Jia Min Chin

**Affiliations:** a Institute of Functional Materials and Catalysis, Faculty of Chemistry, University of Vienna 1090 Vienna Austria jiamin.chin@univie.ac.at; b Vienna Doctoral School in Chemistry, University of Vienna Währinger Straße 42 Vienna 1090 Austria; c Lithoz GmbH Mollardgasse 85a/2/64-69 Vienna 1060 Austria; d Institute of Inorganic Chemistry, Faculty of Chemistry, University of Vienna Währinger Straße 42 Vienna 1090 Austria michael.reithofer@univie.ac.at; e Wolfgang Pauli Institute, Oskar-Morgenstern-Platz 1, Vienna, 1090 Austria

## Abstract

We demonstrate the functionalization of high-temperature sintered, 3D-printed α-alumina ceramics with ZIF-8 and MOF-808 to create robust MOF–ceramic composites. Dense α-alumina sintered at 1450–1650 °C can be directly functionalized despite its low surface hydroxyl density. The composites unite MOF activity with the mechanical strength and design freedom of additive-manufactured ceramics. Using MOF-808, rapid and complete degradation of dimethyl-4-nitrophenyl phosphate (DMNP) was achieved, with cycling tests confirming strong MOF adhesion. Grid-like printed geometries provided high surface area and handling advantages, eliminating centrifugation and filtration required for powders. This work establishes a scalable platform for integrating MOFs with mechanically resilient, architected ceramics for further applications, such as catalysis, separations, and water treatment.

## Abstract 2

3D-printed and densely sintered α-alumina supports with high flexural strength were functionalized with ZIF-8 and MOF-808. The latter was used to catalyze the rapid degradation of nerve-agent simulant dimethyl-4-nitrophenyl phosphate.

## Graphical abstract

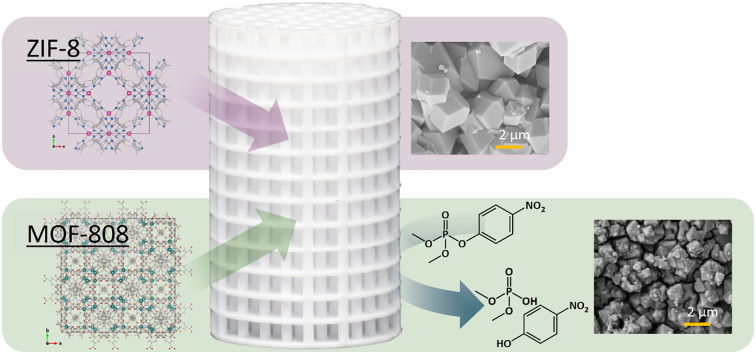

Metal–organic frameworks (MOFs), composed of inorganic metal clusters connected by organic linkers, offer intrinsic porosity, large surface areas, and chemical tunability.^1^ They exhibit remarkable versatility due to the range of metal nodes, organic linkers, and post-synthetic modifications available.^2^ However, processing and scaling MOFs remain challenging. MOF synthesis typically results in loose, crystalline powders that pose handling problems, such as clogging, material loss, and abrasion, leading to increased costs.^3^ To address this, new methods for processing MOFs into more usable forms are being explored, including their incorporation into composite materials and membranes.^4–7^ Ceramics represent an especially promising class of substrates for MOF growth, given their general wear and corrosion resistance, and high hardness. However, unlike plastics, their processability is limited by their lack of solubility, brittleness and extremely high melting points.^8^ Lithography-based ceramic manufacturing (LCM)^9^ is an advanced 3D-printing method that enables the fabrication of complex ceramic shapes unattainable through traditional casting. In this process, ceramic particle suspensions with resin binders are photocured layer-by-layer, followed by debinding to remove the polymer framework and sintering to solidify and strengthen the ceramic structure. Amongst the materials employed, α-alumina is particularly favored due to its low cost, high mechanical stability, and excellent wear resistance but suffers from low surface reactivity, making functionalization challenging. Therefore, studies on the growth of MOFs on alumina substrates are predominantly focused on γ-alumina or alumina sintered at lower temperatures (around 900–1300 °C), which possess higher surface reactivity due to additional hydroxyl groups^10,11^ that promote MOF-binding as well as higher porosity, which offers increased nucleation sites and enhances MOF anchoring.^11–16^ However, alumina prepared this way often comes at the cost of reduced mechanical stability, which impedes its suitability in applications requiring robustness against thermal cycling and turbulent flow, as seen in catalytic processes. Whilst MOF functionalization of high-temperature sintered alumina (1400–1500 °C) has been reported for alumina hollow fiber membranes,^17^ this method relied upon phase inversion to produce microchannels and porosity for MOF anchoring, which can compromise the mechanical integrity of the substrates.

Given the versatility and adaptability of 3D-printed ceramics, and the increasing importance of additive manufacturing as a fabrication tool, we aimed to investigate the use of LCM-fabricated high-temperature sintered alumina as MOF supports, where we optimize the trade-off between reactivity and mechanical strength to create robust MOF-ceramic composites. Conventional grafting methods like sol–gel derived porosity and casting can offer cost- and time savings, but are limited to simple geometries. When substrates bearing more complex geometries, such as internal structures, grids or overhangs, are required, 3D-printing approaches are necessary.^9,18^ In this work, we investigated MOF growth on commercially available α-alumina substrates and evaluated the catalytic activity of the resulting MOF-ceramic composite. LCM-printed α-alumina substrates (Fig. 1 and Fig. S1), sintered at 1450 °C, 1550 °C, and 1650 °C, were utilized as mechanically stable supports for MOF growth. The printed alumina (Al_2_O_3_) ceramics were further functionalized as a proof-of-method, with both an imidazolate-based (ZIF-8) and carboxylate-based (MOF-808) MOF, demonstrating the versatility of this approach (Fig. 2). The MOFs were selected for their mechanical stability, low cost, and resistance to hydrolysis, making them ideal candidates for larger-scale applications. ZIF-8, a Zn-based MOF, has a zeolite-like structure with excellent water stability thanks to its hydrophobicity and strong zinc-imidazolate bonds^19^ and is widely used in water treatment.^20^ MOF-808, a zirconium-based MOF, is primarily used in catalysis,^21,22^ and gas storage and separation.^23^ MOF-808 was further grown onto more intricate 3D-printed α-alumina grids to enhance the accessible surface area and reduce the weight of the fabricated composites for the rapid degradation of the organophosphate dimethyl-4-nitrophenyl phosphate (DMNP), an organophosphate nerve agent simulant and pesticide commonly found in wastewater (Fig. 3).^24^

**Fig. 1:**
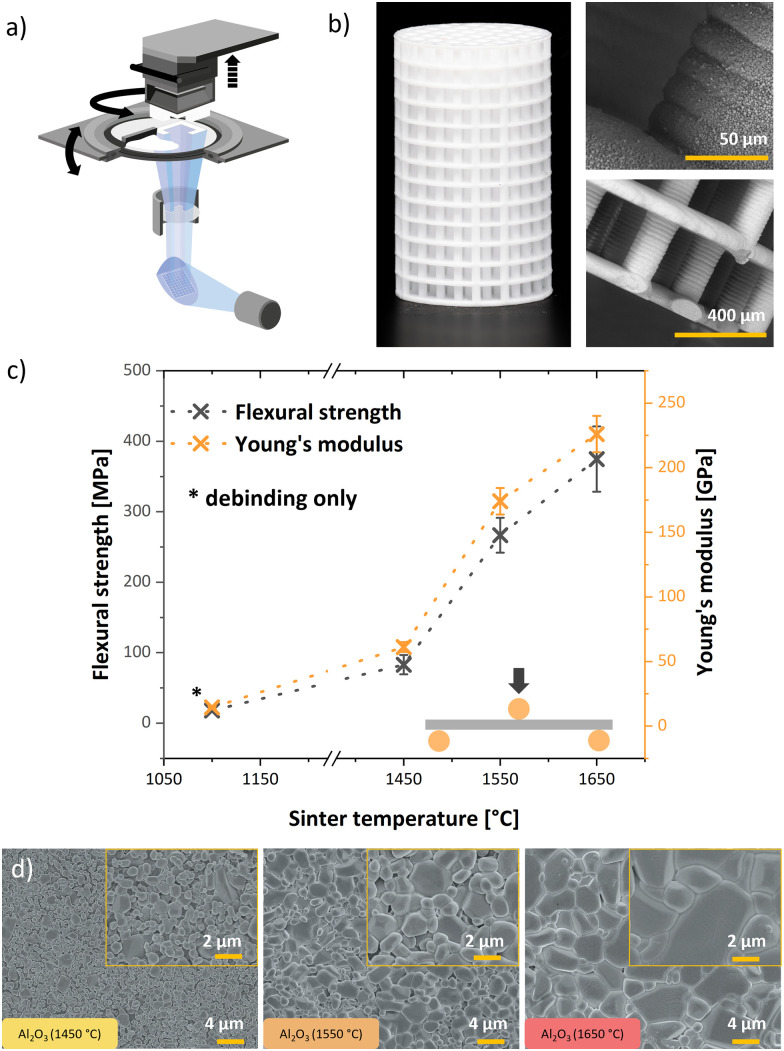
Printing set-up of lithography-based ceramic manufacturing (LCM) by Lithoz GmbH (a), sintered grid-like α-alumina support structure (*Ø* = 2.5 mm, *h* = 5 mm) with MOF-functionalization (b), mechanical properties according to sinter temperature (c) and FE-SEM images of α-alumina sintered at 1450 °C, 1550 °C and 1650 °C (d).

**Fig. 2:**
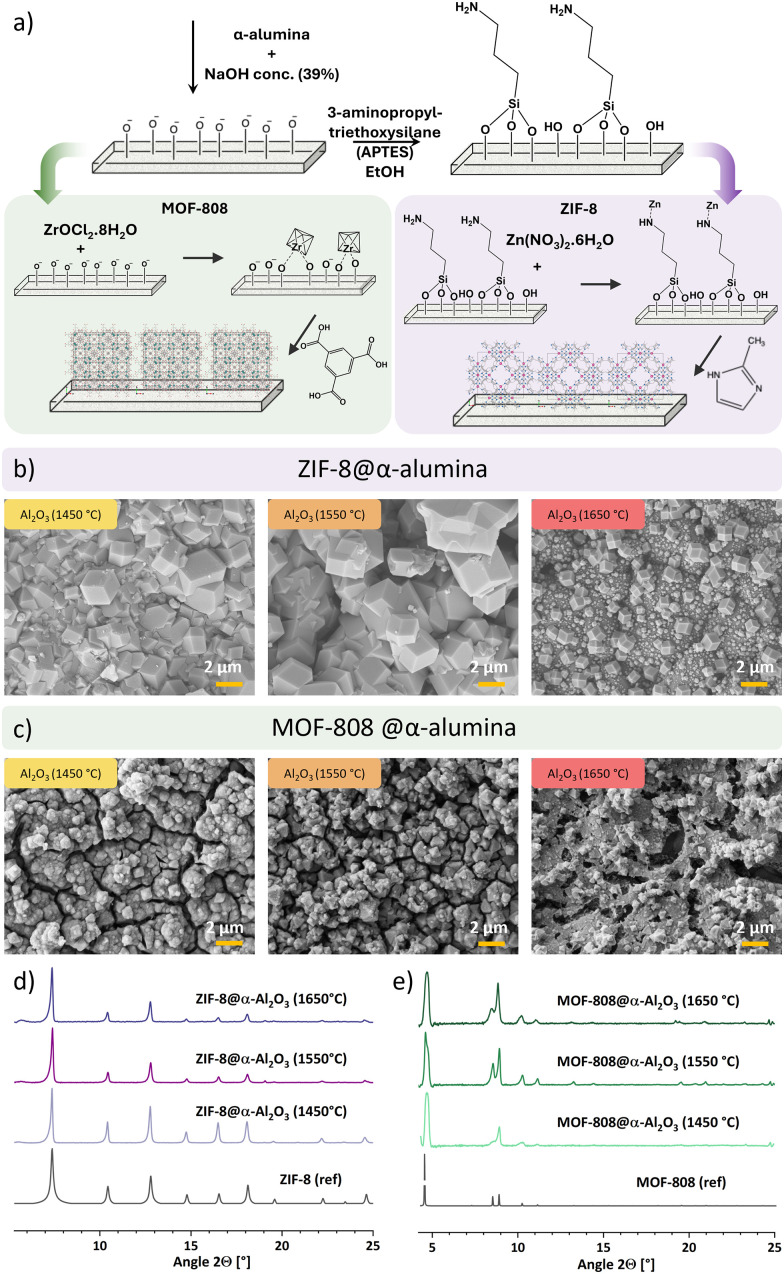
Schematic functionalization of α-alumina with MOF-808 and ZIF-8 (a). FE-SEM images of ZIF-8 (b) and MOF-808 (c) grown onto differently sintered α-alumina. PXRD patterns of ZIF-8@α-alumina (d) and MOF-808@α-alumina (e).

**Fig. 3:**
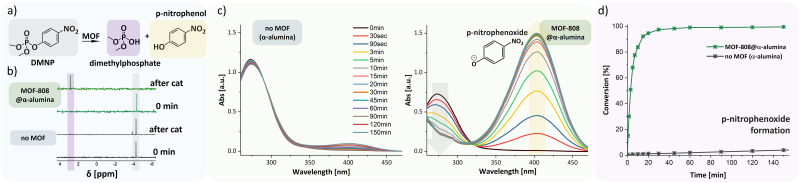
DMNP degradation reaction (a), ^31^P-NMR Spectra of MOF-808@α-alumina and α-alumina before and after catalysis (b), UV-vis absorbance spectra of α-alumina and MOF-808@α-alumina during the reaction (c), and conversion based on *p*-nitrophenoxide formation (d).

The support material for subsequent functionalization was fabricated through LCM-printing, followed by a washing step to eliminate excess slurry. The structures undergo thermal debinding at 1100 °C to remove the organic phase. The employed LCM-printing set-up and complex grid-like structures are shown in Fig. 1a and b. Mechanical properties of 3D-printed alumina sintered at various temperatures were then studied using 3-point bending tests (ASTM C 1161–02c, adapted to the specimen geometry). Results are shown in Fig. 1c, whereby we found that within the sintering temperature range of 1450–1650 °C, the ultimate strength and Young's modulus increases with sinter temperature. Comparing substrates that were only thermally treated at 1100 °C *versus* substrates exposed to a sintering step at 1650 °C, we observe a 20-fold and 16-fold increase in strength and modulus after sintering (Fig. 1). In addition, α-alumina samples sintered at 1550 °C and 1650 °C exhibited a stress/yield-plateau in the stress–strain curves (Fig. S2), likely caused by the denser microstructure featuring larger grains, which promotes stress distribution *via* delayed microcracking due to reduced porosity^25^ and grain boundary sliding.^26^ However, such mechanical advantages are achieved at the expense of their ease of functionalization. Densification with increasing sinter temperatures was confirmed *via* FE-SEM (Fig. 1d). Higher sinter temperatures lead to alumina with larger grain sizes. Sintered alumina also exhibits significantly larger grains compared to supports which only underwent debinding at 1100 °C (Fig. S3).

We subsequently studied the growth of ZIF-8 and MOF-808 (Fig. 2) on the sintered alumina, starting with simple cuboidal substrates bearing flat surfaces for preliminary tests. A schematic of synthesis strategies is shown in Fig. 2a. For both MOFs, pre-etching of α-alumina with NaOH was performed to activate the material by increasing the amount of surface Al–O^−^ groups.^11^ Functionalization with imidazolate-based ZIF-8 was particularly challenging due to the inherent incompatibility between the “soft” imidazole ligands which bind poorly to “hard” aluminum sites of the supports. This was circumvented by functionalization of the supports with 3-aminopropyltriethoxysilane (APTES), which facilitated the growth of ZIF-8 on the alumina. The amine groups of APTES likely coordinate Zn^2+^, allowing for direct growth of a covalently bound ZIF-8 layer.^27,28^ FE-SEM images depicted in Fig. 2b show a well intergrown, continuous coverage of ZIF-8 for α-alumina supports sintered at 1450 °C and 1550 °C. The functionalization of α-alumina sintered at 1650 °C shows decreased coverage, which coincides with our expectations given the lower porosity and fewer anchoring points for MOF-nuclei. Individual crystals rather than an intergrown layer on the smooth substrate are observed.

The carboxylate-based MOF-808 shows good coverage of similar morphologies for all sinter temperatures (Fig. 2c). Oxygen bearing moieties of the etched α-alumina surface bind readily to the oxophilic Zr-atoms^29,30^ whilst the carboxylate-groups from the trimesic acid linkers are expected to bind to Al^3+^. The crystals formed show a degree of polydispersity but adhere well to both the ceramic substrate and each other, forming an intergrown layer on the substrate across all sintering temperatures, covering the α-alumina support. Similarly to ZIF-8, the results for 1450 °C and 1550 °C are quite comparable, while the MOF growth on the most densely sintered support (1650 °C) appears more irregular, with a different, less uniform structure. Both MOFs seem to grow directly from the support with nucleation in pores or on rough spots of the α-alumina leading to a larger size distribution and well anchored crystals (Fig. 2b and c). The samples were further characterized *via* energy dispersive X-ray spectroscopy (EDX) elemental mapping (Fig. S4). Atoms expected for each MOF (Zn or Zr from metal nodes and C from linker molecules) were located at the sites of observed crystals. MOF crystal structures were confirmed using powder X-ray diffraction (PXRD) (Fig. 2d and e).

Having established the parameters for MOF growth on the sintered alumina, grid-like cylindrical supports with large specific surface area (73.3 cm^2^ g^−1^, prior to MOF functionalization) were 3D-printed as support material for the catalysis investigation. Due to the fine structures of the 3D printed cylindrical grids (Fig. 1b), an intermediate sintering temperature of 1550 °C was chosen to impart sufficient mechanical stability for ease of handling, whilst maintaining plentiful anchoring sites for MOF growth.

To demonstrate a potential application of such mechanically robust 3D-printed MOF@ceramic composite materials, MOF-808-functionalized alumina grids were tested for the catalytic degradation of dimethyl-4-nitrophenyl phosphate (DMNP) (Fig. 3), which required vigorous stirring over extended periods. The hydrolyzation of DMNP to dimethyl phosphate and *p*-nitrophenol (*p*-nitrophenoxide in alkaline buffer) is depicted in Fig. 3a. Determination of MOF loading *via* thermogravimetric analysis was not possible due to the low weight percentage of the MOF relative to the alumina supports. Therefore, the MOF loading was estimated based on SEM analysis of the α-alumina supports, approximating the height of the MOF layer to be that of the average crystallite size of the corresponding MOF (ZIF-8, MOF-808) (refer to SI 3a). Catalyst loading was approximated to be 0.211 mg for MOF-808 on each gridded cylindrical substrate.

The reaction progress was followed using ^31^P-NMR (Fig. 3b) to ascertain full conversion of the degradation reaction of the starting material DMNP (*δ* = −4.4 ppm) to the dimethyl phosphate anion (*δ* = 2.8 ppm). Fig. 3b shows a complete absence of the starting material after catalysis using MOF-808@α-alumina. A third peak at *δ* = −3.7 ppm can be observed for the reactions using only α-alumina (no MOF) and is ascribed to another previously reported degradation product of methyl 4-nitrophenyl phosphate (M4NP).^31^*In situ* UV-vis spectroscopy was also carried out (Fig. 3c) to monitor the reaction concentrations of DMNP (Fig. S5) and *p*-nitrophenoxide (Fig. S6), with their respective light absorption bands at 273 and 407 nm.^32^ Reaction progress was also qualitatively observed as a significant color change of the reaction mixture from a clear solution to a deep yellow. In the absorbance spectra shown in Fig. 3c, a rapid conversion of DMNP to *p*-nitrophenoxide can be observed, plateauing after about 60 min, at full conversion to *p*-nitrophenoxide.^21,33,34^ In comparison, at the same time point, the corresponding reaction with unfunctionalized α-alumina reaches a conversion of only 2%, indicating minimal contribution of the substrate to the catalytic reaction. The prepared catalyst was cycled 2 times (Fig. S8), showing full conversion of the nerve agent in both cases, albeit with increased reaction time after the first reaction cycle, which we attribute to partial MOF degradation, a common challenge for MOF catalysts.^35,36^ However, FE-SEM shows that the MOF remains well attached to the support material, even after multiple washings (Fig. S9), indicating the robustness of the functionalization protocol.

Functionalization of high-temperature 3D-printed α-alumina with MOFs yielded robust composites that combined catalytic activity with excellent mechanical stability and handling advantages. Unlike powdered MOFs, which require filtration and often suffer from clogging or material loss, the α-alumina supports enabled easy manipulation and flexibility in form, while allowing complex, lightweight geometries that enhance surface area, fluidic transport, and thermal control. As exemplified by MOF-808 on α-alumina, the composites achieved rapid degradation of the nerve agent simulant DMNP, while the ceramic substrate provides a mechanically resilient, scalable, and easily handled platform. Although catalyst lifetime remains limited by the intrinsic stability of the MOF, strong adhesion of the MOF layer highlights the durability of the composite. 3D-printed ceramics with fine features and complex geometries rely upon high-temperature sintering to impart mechanical robustness and ensure structural integrity, but suffer from limited functionalization possibilities. This work establishes a generalizable approach to MOF functionalization on 3D-printed, high-temperature sintered ceramics. With the design freedom of additive manufacturing and the straightforward synthesis of MOFs, such composites hold significant promise for industrial applications in catalysis, separations, and water treatment, and future efforts will focus on exploiting architectural versatility to tailor long-term performance under demanding conditions.

This project was supported by the Austrian Science Fund (FWF) stand-alone grant DOI 10.55776/P34662 (M. R. R.) and the European Union (ERC Consolidator Grant) DYNAMOF, Grant Agreement 101002176 (J. C.) as well as the Austrian Research Promotion Agency (FFG) grant ReNUSLIC FFG-894592 (J. C.) F. S. thanks Alejandra Durán Balsa for helpful discussions. FE-SEM imaging was performed with the help of Stephan Puchegger at the Faculty Center for Nano Structure Research of the University of Vienna.

## Conflicts of interest

The authors declare no conflict of interest.

## Supplementary Material

MA-006-D5MA01028D-s001

## Data Availability

The data supporting this article have been included as part of the supplementary information (SI). Supplementary information is available. See DOI: https://doi.org/10.1039/d5ma01028d.
