# Supplementary material for: High-temperature sintered 3D-printed alumina as mechanically robust supports for MOF catalysis
Source: Mater Adv. 2025 Oct 10;6(22):8365–9. doi: 10.1039/d5ma01028d (PMC12521964; doi:10.1039/d5ma01028d)
Supplement: MA-006-D5MA01028D-s001 [file MA-006-D5MA01028D-s001.pdf]

Supplementary Information (SI) for Materials Advances.
This journal is © The Royal Society of Chemistry 2025

Supplementary information

High-Temperature Sintered 3D-Printed Alumina as Mechanically Robust Supports for MOF Catalysis

Flora Schöfbeck[a,b], Tanja Eder[a], Wenyi Zeng[a,b], Dominik Brouczek[c], Martin Schwentenwein[c], Youven Benseghir[a], Michael Reithofer*[d] and Jia Min Chin*[a]

a. Department of Functional Materials and Catalysis, University of Vienna, 1090 Vienna, Austria.
b. Vienna Doctoral School in Chemistry, University of Vienna, Währinger Straße 42, Vienna, 1090, Austria.
c. Lithoz GmbH, Mollardgasse 85a/2/64-69, 1060 Vienna, Austria.
d. Department of Inorganic Chemistry, University of Vienna, Währinger Straße 42, Vienna, 1090, Austria

Contents

1. α-Alumina supports 2
   a. Printed supports 2
   b. Mechanical testing (3-point-bending) 2
   c. Scanning electron microscopy (SEM) 3
2. MOF - growth 4
   a. Chemicals 4
   b. Methods 4
   c. Synthesis 4
   d. Energy dispersive X-ray spectroscopy (EDX) 5
3. Catalysis 6
   a. Calculations 6
   b. UV/vis calibration 6
4. References 8

1. α-Alumina supports

a. Printed supports

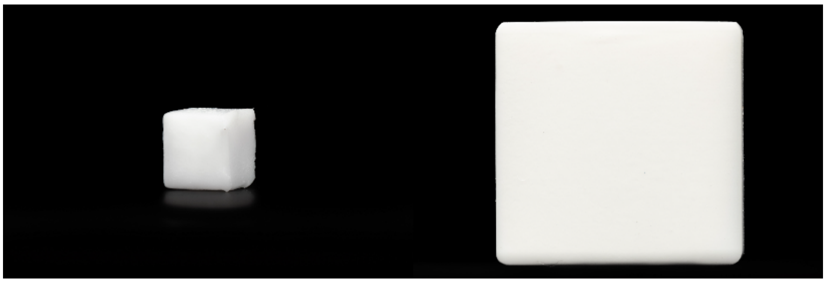

Fig. S1 3D-printed α-alumina pieces (2 x 2 x 2 mm left, 10 x 10 x 2 mm right).

b. Mechanical testing (3-point-bending)

Specimens obtained from sinter temperatures of 1100 °C, 1450 °C, 1550 °C and 1650 °C were printed to afford the cross-sectional dimensions of 1.5 x 2 mm and a minimum length of 25 mm. The cross-sectional thickness of the samples was measured individually with calipers (**Table S1**). The anvils supporting the sample were placed to yield a 20 mm support span and specimens were centrally placed on top. To conduct a test, a corresponding anvil-shaped punch was moved towards the specimen until contact (indicated by a slight increase in load), then moved away by one click and the measurement started. Loading was applied at 0.5 mm/min until failure. The ultimate strength was calculated as the maximum recorded force divided by the area for each test and averaged. The elastic modulus was obtained from the slopes of the linear portion of the stress strain curves for each sample and averaged.

Table S1 Average dimensions of α-alumina samples for 3-point bending mechanical testing.

Sinter Temperature [°C]	Mean dimensions [mm]		Std. Deviation	
	a	b	a	b
1100	1.575	2.04917	0.01087	0.00669
1450	1.63333	2.13667	0.01303	0.02462
1550	1.57375	2.04875	0.00518	0.00641
1650	1.603	2.117	0.0283	0.01418

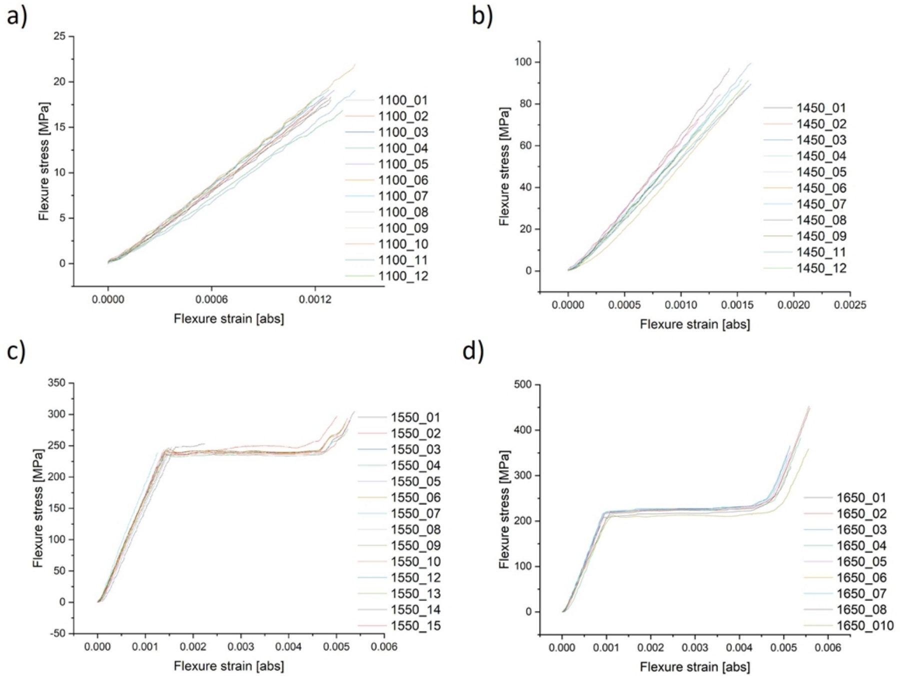

Fig. S2 Stress-strain curves of α-alumina samples sintered at 1100 °C (a), 1450 °C (b), 1550 °C (c) and 1650 °C (d).

c. Scanning electron microscopy (SEM)

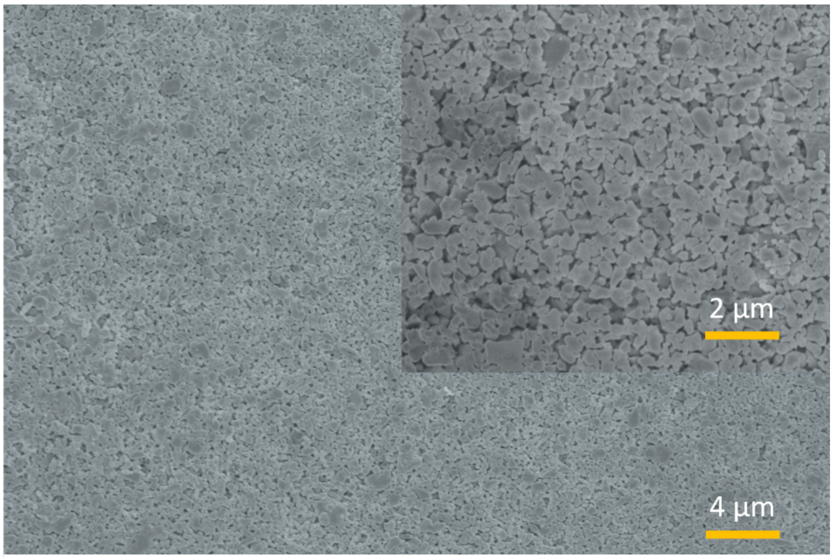

Figure S 3 SEM images of α-alumina sintered at 1100 °C.

2. MOF - growth

a. Chemicals

Chemicals used are 2-methylimidazole (2-MIm, Alfa Aesar), trimesic acid (Alfa Aesar, 98%), Zn(NO3)$_2$.6H_2O (Sigma-Aldrich, 98%), $ZrOCl_2$.8H_2O (Alfa Aesar, 98%), 3-aminopropyltriethoxysilane (APTES, Sigma Aldrich), conc. NaOH (39%, Fisher), acetic acid (Thermo Fisher Scientific, 99.8%), methanol (Honeywell, 99.9%), ethanol (96%), H_2O (MilliQ) and DMF (Chem- solute, 99%). All chemicals were used without further purification. For catalytic studies, dimethyl-4-nitrophenyl phosphate (DMNP, Sigma Aldrich) and 4-ethylmorpholine (Alfa Aesar) were purchased.
The 3D-printed α-alumina supports were provided by Lithoz GmbH. All samples are printed from LithaLox350 using LCM technology (**Fig. 1a**) and subsequently sintered at different temperatures of 1450 °C, 1550 °C and 1650 °C, respectively. Samples were prepared as monolithic structures with dimensions 1 x 1 x 0.2 cm (**Fig. S1**) and as more complex cylindrical grid support structures (**Fig. 1b**).

Monoliths created with LCM printing technique were fabricated using a CeraFab L30 3D printer with 40 µm resolution (40 x 40 μm pixel size with a total of 1920 x 1080 pixels) and consequently have features at micrometer resolution.[1-3]

b. Methods

Scanning electron microscopy (SEM) and energy dispersive x-ray (EDX) spectroscopy were performed using a Hitachi TM4000 Tabletop Scanning Electron Microscope and a Zeiss Supra 55 VP Field Emission Scanning Electron Microscope with an EDX unit from oxford instruments. Powder X-ray diffraction (PXRD) was measured with an Empyrean Panalytical diffractometer in reflection-transmission spinner configuration (Cu Kα λ=1.45 Å). An Agilent Cary 60 UV-vis spectrometer and ^{31}P-NMR were used to track DMNP degradation. All ^{31}P-NMR measurements were recorded at the NMR center of the Faculty of Chemistry on a Bruker BioSpin AV NEO 500 with a frequency for ^{31}P of 202.44 MHz. Three point bending tests were conducted in an Instron universal testing machine equipped with a 1 kN load cell and a 5 kN 3-point flexure fixture. DIN EN 843-1:2008-08/ ASTM C 1161 – 02c was adapted regarding the specimen sample geometry (**Table S1**). Results were further subjected to a One-way ANOVA to ensure statistical significance.

c. Synthesis

Growth of ZIF-8 on sintered α-alumina. After an etching-procedure of 2 h in conc. NaOH (39 w%) the supports were immersed into an APTES/EtOH solution (v:v 1:50) for 2 h at room temperature. For the MOF synthesis, the supports were soaked in the metal-salt solution (0.174 mmol, 33 mg $Zn(NO_3)_2$.6H_2O in 5 mL H_2O) for 2 h. Subsequently, the ligand solution (8.2 mmol, 675 mg 2-MIm in 5 mL H_2O) was slowly added dropwise. The mixture was kept at 50 °C for 24 h after which the support was washed 3x with MeOH or EtOH.
Growth of MOF-808 on sintered α-alumina. Supports were etched in conc. NaOH (39 w%) for 2 h. The ligand H_3BTC (trimesic acid 0.1 mmol, 21 mg) and the metal precursor $ZrOCl_2$.8H_2O (0.3 mmol, 97 mg) were each dissolved in 4.5 mL of solvent consisting of DMF and formic acid (v:v 1:1) respectively. Supports were soaked in the metal solution for 1 h, then the ligand solution was added. The reaction was transferred to a hydrothermal vessel, sealed, and heated at 130 °C for 48 h. After synthesis the supports were thoroughly washed with fresh DMF and acetone. A solvent exchange using fresh acetone was performed over 3 days. The synthesis of both MOFs is schematically depicted in **Fig. S4**.

d. Energy dispersive X-ray spectroscopy (EDX)

Two different samples of ZIF-8@α-alumina were prepared, one with NaOH etching (low coverage) of the support and one with APTES pretreatment (high coverage) of the support. The aluminum (Al) atom mapping density is much lower for the high MOF coverage sample as the support is effectively shielded by the Zn-MOF. Additionally, the Zn- and C- density (ZIF-8), as well as Zr- and C-density (MOF-808) which stems from the metal and linker of the MOF is elevated in locations where the crystalline particles are clearly visible via SEM.

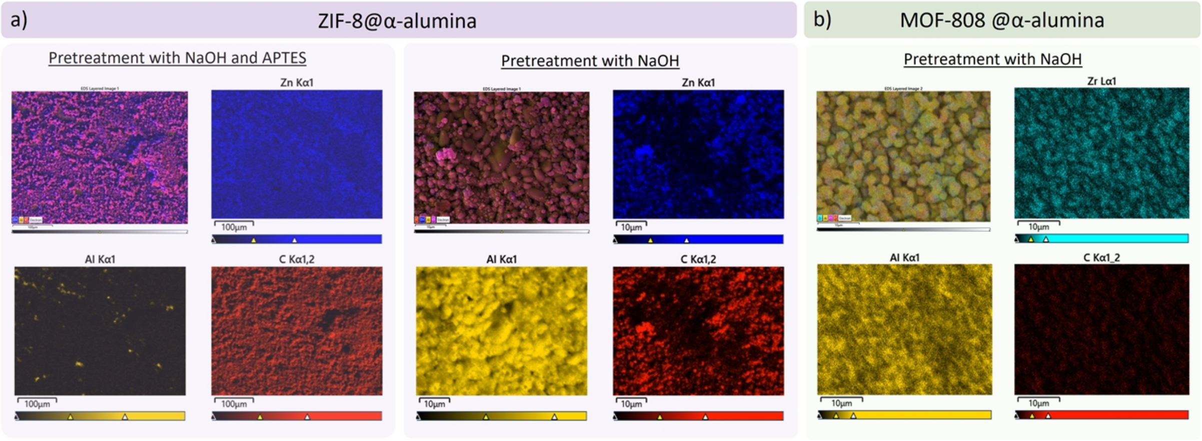

Fig. S4 EDX maps of ZIF-8@α-alumina and MOF-808@α-alumina (b).

3. Catalysis

a. Calculations

Due to the very small amounts of MOF present on the ceramic supports, mass determination of the MOF layer by sample weighing was not possible. Therefore, a rough approximation to estimate the synthesized product was performed to design the catalytic study. In the following, the approximation steps are explained.

All parameters for the calculations are listed in **Table S2** and **Table S3.** The surface of the grid-like cylindrical alumina supports is 2.21 cm^2 and the height of the MOF layer was estimated with the help of SEM images with the assumption that a monolayer was formed. The mass of one singular unit cell m_{UC} was calculated via division by Avogadro's number k. The monolayer volume V_{ML} was calculated multiplying the surface area A_{sf} with the crystal height h_{crys}. The number of unit cells per monolayer N_{UC} was obtained via division of the monolayer volume by the volume of one unit cell V_{UC}. The mass of the MOF m_{MOF} on the ceramic support results from multiplying the mass of one singular unit cell with the number of unit cells.

For ZIF-8 the same calculations were performed using the corresponding crystal system. Parameters are listed in **Table S2** and refer to reported ZIF-8 structures.[4] In the ZIF-8 structure one unit cell contains 12 formula units and the estimated crystal height is 2 µm. The mass of the ZIF-8 MOF on the support is therefore approximated as 0.368 mg.

Table S2 ZIF-8 crystal lattice data.

space group	a [Å]	α [°]	V_{UC} [Å^3]	formula	M [g.mol^{-1}]	M_{UC} [g.mol^{-1}]
$I\bar{4}3m$	16.8	90	4767.3	$C_6H_6N_4$	199.5	2394.2

For MOF-808 the same calculations were performed using the corresponding crystal system. Parameters are listed in **Table S3** and refer to reported MOF-808 structures.[5] In the MOF-808 structure one unit cell contains 12 formula units and the estimated crystal height is 1 µm. The mass of the MOF-808 on the support that stems from calculations is 0.211 mg.

Table S3 MOF-808 crystal lattice data.

space group	a [Å]	α [°]	V_{UC}	Formula [Å^3]	M [g.mol^{-1}]	M_{UC} [g.mol^{-1}]
$Fd\bar{3}m$	35.1	90	43156	$C_{24}H_6O_{44.35}Zr_6$	1551.2	24819.4

b. UV-vis calibration

Calibrations of DMNP and the p-nitrophenoxide were carried out to obtain calibration curves to ensure concentration accuracy. All corresponding absorbance spectra and corresponding Linear regressions are depicted in **Fig. S6** and **Fig. S7**.

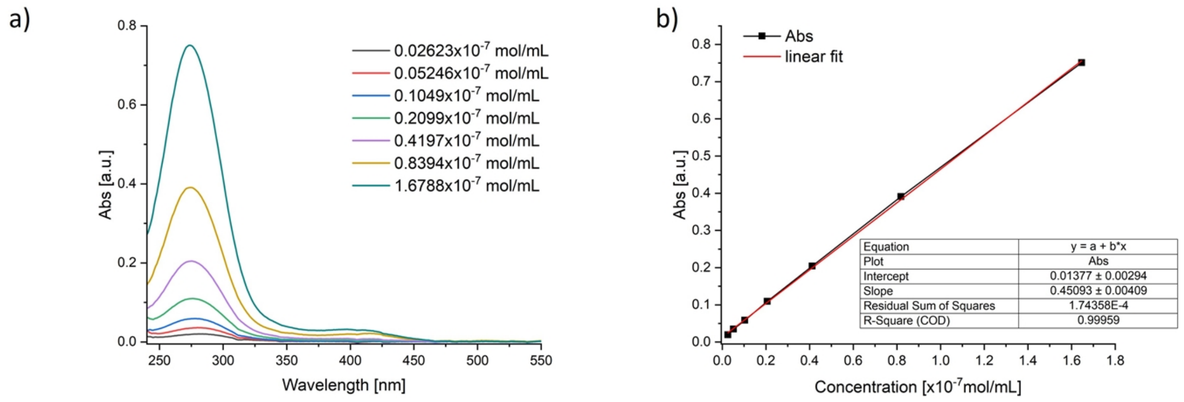

Fig. S5 UV-vis calibration absorbance spectra (a) and linear calibration curve (b) of DMNP.

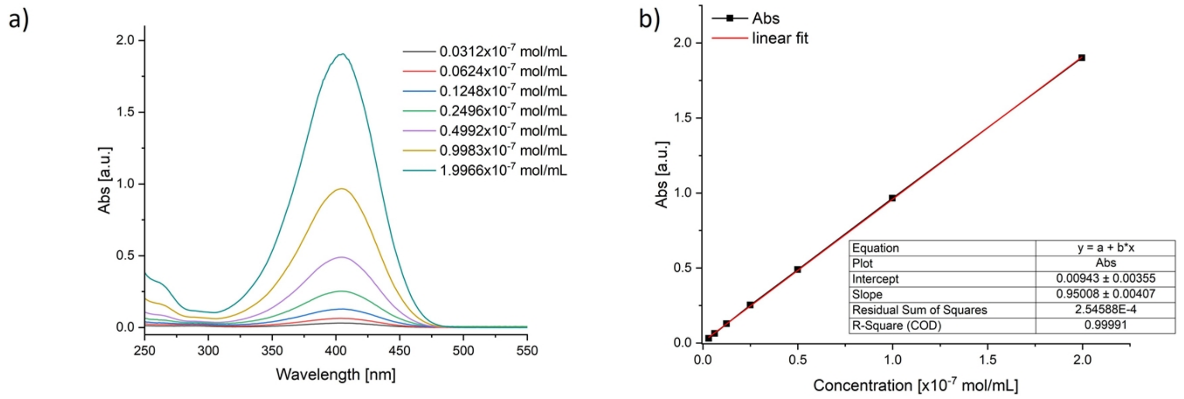

Fig. S6 UV-vis calibration absorbance spectra (a) and linear calibration curve (b) of p-nitrophenoxide.

For the degradation study, DMNP (0.4 μL) was added to 10 mL of *N*-ethylmorpholine (0.45 M, 10% D_2O). 850 μL of this suspension were transferred to a screw top quartz-cuvette. Catalysts were activated at 110°C overnight and soaked in water for 2h. The alumina support was added to the reaction mixture and the catalytic reaction was performed with and without MOF present.
Catalysis was performed over the period of 70 h for plain α-alumina with conversion reaching 25%. Absorbance spectra as well as conversion graphs are shown in **Fig. S7**.

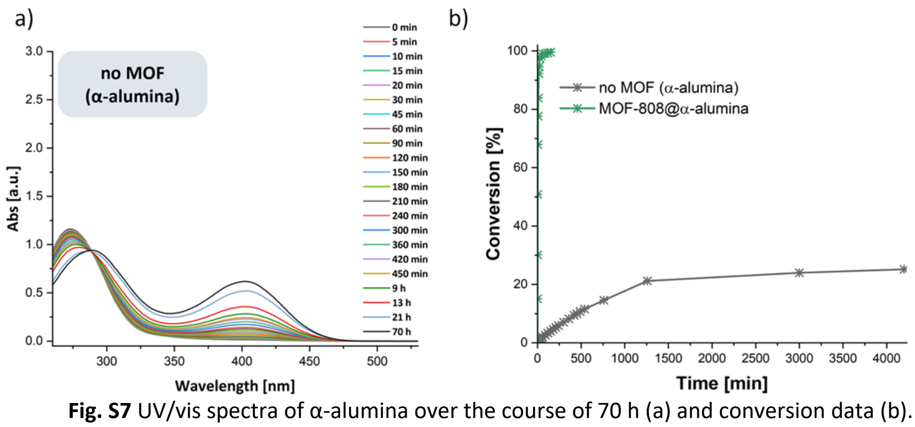

Fig. S7 UV/vis spectra of α-alumina over the course of 70 h (a) and conversion data (b).

The degradation reaction was performed three times with previously described activation between each cycle (**Fig. S9**).

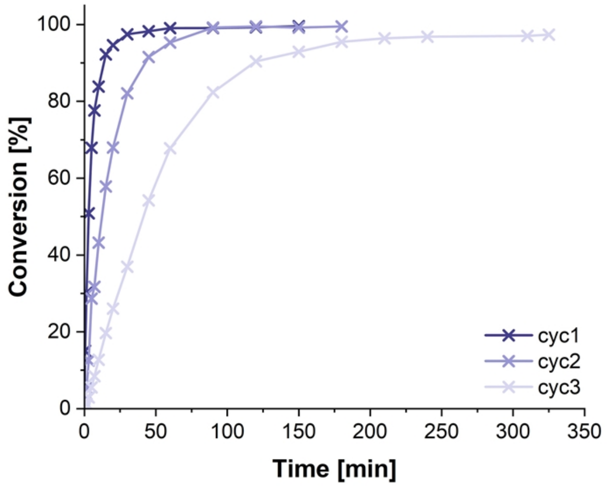

Fig. S8 Cycling of MOF-808@α-alumina.

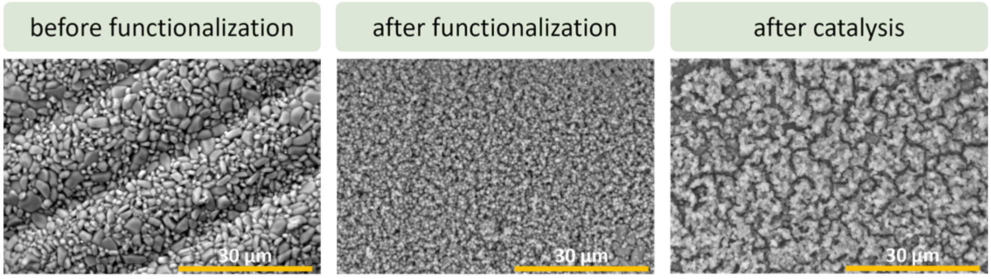

Fig. S9 SEM images of the α-alumina support grids before and after functionalization with MOF-808 (left, middle) and after catalysis (right).

4. References

1. A. A. Altun, T. Prochaska, T. Konegger and M. Schwentenwein, *Appl. Sci.*, 2020, **10**, 996.
2. U. Scheithauer, E. Schwarzer, T. Moritz and A. Michaelis, *J. Mater. Eng. Perform.*, 2018, **27**, 14-20.
3. A. Zocca, P. Colombo, C. M. Gomes and J. Günster, *J. Am. Ceram. Soc.*, 2015, **98**, 1983-2001.
4. O. Karagiaridi, M. B. Lalonde, W. Bury, A. A. Sarjeant, O. K. Farha and J. T. Hupp, *J. Am. Chem. Soc.*, 2012, **134**, 18790-18796.
5. H. Furukawa, F. Gandara, Y. B. Zhang, J. Jiang, W. L. Queen, M. R. Hudson and O. M. Yaghi, *J. Am. Chem. Soc.*, 2014, **136**, 4369-4381.
